# Extent, Awareness and Perception of Dissemination Bias in Qualitative Research: An Explorative Survey

**DOI:** 10.1371/journal.pone.0159290

**Published:** 2016-08-03

**Authors:** Ingrid Toews, Claire Glenton, Simon Lewin, Rigmor C. Berg, Jane Noyes, Andrew Booth, Ana Marusic, Mario Malicki, Heather M. Munthe-Kaas, Joerg J. Meerpohl

**Affiliations:** 1 Cochrane Germany, Medical Center—University of Freiburg, Faculty of Medicine, University of Freiburg, Freiburg, Germany; 2 Global Health Unit, Knowledge Centre for the Health Services at the Norwegian Institute of Public Health, Oslo, Norway; 3 Global Health Unit, Knowledge Centre for the Health Services at the Norwegian Institute of Public Health, Oslo, Norway and Health Systems Research Unit, South African Medical Research Council, Cape Town, South Africa; 4 Unit for Social Welfare Research, Knowledge Centre for the Health Services at the Norwegian Institute of Public Health, Oslo, Norway and the Department of Community Medicine, University of Tromso, Tromso, Norway; 5 School of Social Sciences, Bangor University, Bangor, United Kingdom; 6 School of Health and Related Research, University of Sheffield, Sheffield, United Kingdom; 7 Cochrane Croatia and Department of Research in Biomedicine and Health, University of Split, School of Medicine, Split, Croatia; 8 Unit for Social Welfare Research, Knowledge Centre for the Health Services at the Norwegian Institute of Public Health, Oslo, Norway; 9 Centre de Recherche Épidémiologie et Statistique Sorbonne Paris Cité–U1153, Inserm / Université Paris Descartes, Cochrane France, Hôpital Hôtel-Dieu, Paris, France; Stanford University, UNITED STATES

## Abstract

**Background:**

Qualitative research findings are increasingly used to inform decision-making. Research has indicated that not all quantitative research on the effects of interventions is disseminated or published. The extent to which *qualitative* researchers also systematically underreport or fail to publish certain types of research findings, and the impact this may have, has received little attention.

**Methods:**

A survey was delivered online to gather data regarding non-dissemination and dissemination bias in qualitative research. We invited relevant stakeholders through our professional networks, authors of qualitative research identified through a systematic literature search, and further via snowball sampling.

**Results:**

1032 people took part in the survey of whom 859 participants identified as researchers, 133 as editors and 682 as peer reviewers. 68.1% of the researchers said that they had conducted at least one qualitative study that they had not published in a peer-reviewed journal. The main reasons for non-dissemination were that a publication was still intended (35.7%), resource constraints (35.4%), and that the authors gave up after the paper was rejected by one or more journals (32.5%). A majority of the editors and peer reviewers “(strongly) agreed” that the main reasons for rejecting a manuscript of a qualitative study were inadequate study quality (59.5%; 68.5%) and inadequate reporting quality (59.1%; 57.5%). Of 800 respondents, 83.1% “(strongly) agreed” that non-dissemination and possible resulting dissemination bias might undermine the willingness of funders to support qualitative research. 72.6% and 71.2%, respectively, “(strongly) agreed” that non-dissemination might lead to inappropriate health policy and health care.

**Conclusions:**

The proportion of non-dissemination in qualitative research is substantial. Researchers, editors and peer reviewers play an important role in this. Non-dissemination and resulting dissemination bias may impact on health care research, practice and policy. More detailed investigations on patterns and causes of the non-dissemination of qualitative research are needed.

## Background

Decision-makers are increasingly interested in understanding the views, behaviors and experiences of health service users, health care providers and other stakeholders [1–3]. An understanding of these groups’ beliefs about health issues in general, as well as of their experiences of and preferences for specific health care management strategies such as treatment options, goes beyond what can be expressed quantitatively and is highly relevant [4]. Such understanding can help decision-makers assess the acceptability of interventions and diagnostic tests, the feasibility of implementing interventions, and the importance people place on different health outcomes. Qualitative research is an appropriate way of achieving this understanding and qualitative research findings are therefore increasingly used to inform decision making [5, 6]. In this context, we define qualitative research as those studies that used a qualitative method of data collection and a qualitative method of data analysis.

Accordingly, researchers are increasingly synthesizing evidence from qualitative research studies [7] in qualitative evidence syntheses (QES) [8]. These syntheses can be used to summarize current knowledge and to support decision-makers in making choices. QES can also be used to inform the development of fields of research, for instance by contributing to empirical generalizations and building theory through providing an overview of what is going on in the field [9].

When undertaking a QES, researchers aim to gain an overview of existing research. One barrier to identifying all primary research relevant to a review question is the failure of primary authors to publish their study or to make their research available in other ways. In the field of quantitative research, studies have indicated that not all studies on the effectiveness of interventions are published or otherwise made available to decision-makers [10, 11]. Specifically, studies showing that an intervention had little or no effect are less likely to be made available than studies showing beneficial or harmful effects, leading to systematic biases in estimates of effectiveness [10, 12]. This problem is widely recognized within quantitative research environments and commonly referred to as publication bias or, more broadly, dissemination bias, and is also likely to be present in qualitative research [13].

Any study that is not available to the scientific community is a waste of resources. Firstly, unpublished research on humans is a deceit of research participants who contributed their personal time and resources to add to the success of the study. Furthermore, a sizeable proportion of studies are funded by public resources. Conducting a study with public funding and not disclosing or not publishing the findings is a waste of public money. The irretrievability of these studies in systematic searches might lead to bias in QES. Furthermore, empirical research has been conducted on the effects of non-dissemination of quantitative research and it is documented that dissemination bias usually leads to an overestimation of the effect in systematic reviews of quantitative studies on health interventions [14, 15]. This can lead to inappropriate and even harmful decisions in health care.

The extent to which *qualitative* researchers also systematically underreport or fail to publish certain types of research findings, and the impact this may have on our understanding of a phenomenon, has received little attention to date. To operationalize this issue we have defined dissemination bias in qualitative research as a systematic distortion of the phenomenon of interest due to selective dissemination of studies or individual findings of studies [16]. As QES become more common, we need to explore the issue of dissemination bias and the extent to which this needs to be taken into account when we assess how much confidence we have in findings from QES. The approach for the assessment of the confidence in findings from QES and the role dissemination bias may play is described in Box 1.

**Box 1. Dissemination bias in the GRADE-CERQual approach**

The GRADE-CERQual approach [16] is designed specifically to assess how much confidence to place in findings from reviews of qualitative evidence. Confidence in the evidence is defined as the extent to which the review finding is considered a reasonable representation of the phenomenon of interest. CERQual’s assessment of confidence for individual review findings is–as of now–based on four components: the methodological limitations of the studies contributing to a review finding, the relevance to the review question of the studies contributing to a review finding, the coherence of the review finding, and the adequacy of data supporting a review finding. Concerns in relation to these components may lower our confidence in a review finding.

Dissemination bias is not currently included in the GRADE-CERQual approach because empirical evidence is very limited on its extent in qualitative research as well as on its impact on findings of QES. Hence, further research is needed to establish the extent of dissemination bias in qualitative research.

Our study has two aims: first, we aim to explore stakeholders’ views and experiences of and reasons for their own and others’ non-dissemination of qualitative research studies and individual findings. Second, we want to explore stakeholders’ views and experiences regarding the issue of dissemination bias in qualitative research and to elicit their views on interventions that might decrease dissemination bias in qualitative research.

## Methods

We used a cross-sectional survey approach to gather data regarding non-dissemination and dissemination bias in qualitative research from qualitative researchers, authors of QES, and peer reviewers and editors of scientific journals publishing qualitative research.

### Survey

We adapted a survey [17] that was originally used to explore dissemination bias in quantitative research. First, we modified the survey after input from researchers with expertise in qualitative and quantitative research. We then revised according to extensive group discussions. We piloted the survey among eight experienced qualitative researchers to test the comprehensibility and comprehensiveness of the questions and answer options. We then made changes to the wording and structure of the survey in response to feedback from the eight researchers.

The survey had five main parts, with each part addressing a different group of participants:

Part 1: researchers with experience in conducting qualitative research
Part 2: authors of QES
Part 3: editors of scientific publishing outlets, such as journals or the Cochrane Library
Part 4: peer reviewers of manuscripts submitted to these outlets
Part 5: all study participants

Respondents could have multiple roles and answer more than one part of the questionnaire (S1 File).

Overall, the survey included 30 questions. There were 20 multiple-choice questions (“check one”/“check all that apply”), four questions with matrix tables (1–5 scale ranging from “strongly agree” to “strongly disagree” or from “always” to “never”) and one question with a drop-down selection. Five of the questions allowed free text responses only. For nine questions, participants could insert free text in addition to a multiple-choice selection in order to elaborate on the answers given to the closed question. We applied a survey-logic with gatekeeper questions and logical skips in order to display only relevant questions to the respective participants. The final survey was delivered online using Survey Monkey (www.surveymonkey.com).

We sent invitations to participate in the survey via e-mail on 13^th^ August 2015 and one reminder two weeks after the commencement of the survey, on 26^th^ August 2015. The survey closed on 6^th^ September 2015.

Respondents were asked to exclude research projects that they conducted/supervised in the context of scholarly degrees, such as masters’ or PhD theses. While we recognize and appreciate the quantity and quality of these, we excluded student projects from other research projects because we assumed that they might not primarily be intended for public dissemination, but rather for academic graduation.

### Recruitment of Survey Addressees

In order to identify suitable participants with experience in qualitative research, we searched for authors of published qualitative studies. We used a validated search strategy for qualitative studies in Ovid MEDLINE [18]. Before use, we further validated the search strategy by title and abstract screening of a random sample (generated using www.random.org) of 200 search results found with the search strategy in Ovid MEDLINE. This validation screening resulted in 195 included records and five excluded records. The excluded records either did not report on a research study or reported a study that did not use a qualitative methodology. Therefore, we concluded that the search strategy fit our purpose.

We conducted the search in Ovid MEDLINE on 14^th^ July 2015 and limited the search period to papers published in the previous six months, from 12^th^ January to 12^th^ July 2015. We set this time limit in order to reach researchers who could easily recall a recent qualitative study and to gather as many valid e-mail addresses as possible. We extracted available e-mail addresses automatically from the search results using a programming script (Perl). The search in Ovid MEDLINE resulted in 3965 references with 1813 tagged e-mail addresses. A single e-mail address was tagged in 1594 publications, whereas more than one e-mail address was included in 149 publications. An invitation to participate in the survey was sent to all of the 1813 retrieved e-mail addresses. The invitation could not be delivered to 2.6% of the addresses. Four addresses were invalid and our invitation was returned. One of them included updated contact details of the author and we forwarded the invitation to the new e-mail address.

We also invited participants through various relevant mailing lists which we knew to contain researchers who were involved in qualitative research (see Appendix). For each mailing list, we identified an individual in charge or included in the list, respectively, beforehand and asked them to distribute our invitation to all e-mail addresses contained in the mailing list. Altogether, the utilized mailing lists included approximately 12,981 recipients. All mailing lists are open for new members and do not have specific entry requirements or charges for their members. Lastly, we asked participants to circulate the invitation within their own professional networks. It was not possible to ascertain the exact number of people reached by our invitation as distributed by the latter two approaches and we therefore cannot calculate total response rates.

### Data extraction, analysis and reporting

All available responses were included in the final analyses. We analyzed data from closed questions using descriptive statistics. We reported the results based on the number of responses to each question separately and calculated proportions accordingly. We used R: A Language and Environment for Statistical Computing [19] to calculate Spearman’s ρ in order to determine associations between variables.

For the free text responses, we conducted an inductive thematic analysis [20] where we organized the answers into themes. We went back and forth between themes and the free text answers, allowing new themes to emerge and making sure that all key themes were identified.

### Ethics approval and data protection

The local ethics committee of the University of Freiburg approved the study. We discussed and clarified data protection concerns with the data protection officer of the Medical Center—University of Freiburg and the director of the ethics committee. We collected no personal data from the participants: responses were anonymous and could not be traced back to individuals and data were analyzed in aggregate form.

At the end of the survey we invited participants to join a mailing list so that participants who were interested in the topic could be contacted and results shared. We collected e-mail addresses separately from the survey responses so that a link between the two could not be established.

## Results

Overall, 1032 people initiated the survey of whom 73.3% completed the survey. The 1813 invitations sent to authors of qualitative research identified through the literature search resulted in a response rate of 4.5% (n = 81), of whom 81.5% completed the survey. The invitations sent to authors of qualitative research identified through the literature search constituted 7.8% of our sample, whereas the invitations sent through mailing lists and snowball sampling constituted 92.2% of our sample. Overall, 96.3% of the respondents identified as researchers, 83.8% identified as peer reviewers, and 16.0% identified as editors. There was overlap as many respondents had multiple roles. Their main characteristics are shown in Table 1.

**Table 1:** Characteristics of participants.

		% (n)
**Role (multiple answers possible)**	Researcher	96.3 (994)
	Editor	16.0 (133)
	Peer reviewer	83.8 (691)
**Gender (N=1032)**	Female	58.1 (600)
	Male	15.3 (158)
	Other	0.01 (9)
	No response	25.7 (265)
**Age (years) (N=739)**	Median = 49.0	
	Interquartile range = 16	
**Main region of work (N=1032)**	Europe	48.0 (363)
	North America	27.6 (209)
	South America	2.2 (17)
	Africa	3.7 (28)
	Asia	7.1 (54)
	Australia and Oceania	10.0 (76)
	No response	27.6 (285)
**Language that findings are mainly communicated in (multiple answers possible) (N=757)**	English	98.5 (743)
	Spanish	4.6 (35)
	French	4.5 (34)
	Dutch	3.2 (24)
	Swedish	3.1 (23)
	Norwegian	2.9 (22)
	German	2.7 (20)
	Portuguese	2.5 (19)
	Finnish	1.6 (12)
	Italian	1.5 (11)
	Persian (Farsi), Turkish	1.2 (9) each
	Danish	0.9 (7)
	Chinese	0.8 (6)
	Japanese	0.5 (4)
	Arabic, Brazilian (not further specified), Slovenian, Thai	0.3 (2) each
	Australian (not further specified), Hebrew, Hindi Marathi, Icelandic, Korean, Malay, Native Fijian Language, New Zealandic (not further specified), Polish, Serbian, Swahili, Welsh, Xitsonga	0.1 (1) each

### Extent of non-dissemination of qualitative studies

Of the 859 participating researchers, 68.1% said they had conducted at least one qualitative study that had not been published in a peer-reviewed journal and 11.5% of them reported that they had published fewer than half of the qualitative studies that they had conducted (see Table 2). Further, 51.7% of researchers stated that they had conducted at least one study that was not published in any publicly accessible format, while 5.6% reported that more than half of their qualitative studies were not publicly accessible. The number of studies conducted by a researcher (Table 3) was not associated with the number of studies that they had published (p = 0.9).

**Table 2:** Qualitative studies published in a peer-reviewed journal.

Proportion of studies	Proportion of researchers who did NOT publish studies in a peer-reviewed journal (by categories in column 1) % (n) (N = 859)
0%	31.9 (274)
1–20%	33.9 (291)
21–40%	12.6 (108)
41–60%	10.1 (87)
61–80%	4.9 (42)
81–100%	6.6 (57)

**Table 3:** Qualitative studies completed by the participating researchers.

Number of completed studies	Proportion of researchers who completed studies (by categories in column 1) % (n) (N = 961)
0	4.8 (46)
1–5	55.1 (529)
6–10	20.9 (201)
11–15	8.7 (84)
16 or more	10.5 (101)

Researchers participating in the survey estimated that other researchers had disseminated a lower proportion of qualitative studies than was the case for these participating researchers themselves. The discrepancies in the estimated proportion of own and others’ published studies are listed in Table 4. We saw the most pronounced difference for the category “All studies have been published in a publicly accessible format”: 48.3% of the researchers said that all of their own qualitative studies were publicly accessible, whereas only 1.7% of them thought that other researchers had disseminated all their studies in a publicly accessible way. On the contrary, similarly low proportions of respondents reported that 81–100% of their own (2.8%) or others’ (1.2%) studies were not published in a publically accessible format.

**Table 4:** Comparison of researchers’ own non-dissemination of studies and their estimates of non-dissemination of studies by other researchers.

Proportion of studies NOT published in any publicly accessible format	Proportion of own studies NOT published in a publicly accessible format, as estimated by participating researchers (by categories in column 1) % (n) (N = 859)	Proportion of other researchers’ studies NOT published in a publicly accessible format, as estimated by participating researchers (by categories in column 1) % (n) (N = 843)
0%	48.3 (415)	1.7 (14)
1–20%	32.8 (282)	19.2 (162)
21–40%	7.3 (63)	24.1 (203)
41–60%	5.9 (51)	20.2 (170)
61–80%	2.8 (24)	7.9 (67)
81–100%	2.8 (24)	1.2 (10)
Not sure	Not applicable	25.7 (217)

### Reasons for non-dissemination of qualitative studies as reported by researchers

The most common reason for non-dissemination given by 412 researchers was that publication was still planned (35.7%). Resource constraints were reported as playing a role for 35.4% of respondents while rejection of a paper by one or more journals was reported as a reason for 32.5% of respondents to abandon publication (multiple responses were possible). Only a few respondents reported that the non-dissemination of an entire study was linked to the findings of the study. Specifically, findings that did not confirm the authors’ assumptions and concepts (2.4%) or findings that were controversial in relation to current knowledge in the field (2.7%) were rarely mentioned as reasons for non-dissemination of a qualitative study. In their free text responses, participants indicated that the publication processes of journals hindered the publication of qualitative studies in a range of ways. One respondent summed up the views of many other respondents, stating: “Editors of peer-reviewed journals themselves do not understand what qualitative research is and often reject articles for reasons such as "too few interviewees" when you have 90 interviews analyzed. The number of words in peer review journals do[es] not fit with qualitative research.” In addition, resource constraints such as time, staff and expertise were reported to make the write-up and dissemination of qualitative studies challenging. As one respondent wrote: “We simply did not have enough qualitative researchers to ensure a good publication […]”. Respondents also mentioned failure to obtain approval for the study by an ethics review board as a reason for non-dissemination.

When asked about further reasons for non-dissemination that were not primarily influenced by the authors themselves, 55.6% selected the option “not applicable”. However, 26.7% reported that they had not submitted the paper because they thought that journals were unlikely to accept their study for publication. Furthermore, a small proportion of researchers reported that sponsors of the research study were either not interested in publishing the findings (6.3%), or had actively prevented the authors from publishing the study (3.6%). The existence of other publications with similar findings to their own was mentioned as a reason for non-dissemination by 5.1% of the respondents. In their free text responses, researchers provided more information on how funders were involved in the publication process of their studies. They reported that funders were “[…] quite stingy in the amount of time/resources they allow for dissemination […]” and that the funders required the researchers to move to other project tasks. This left researchers with no time to produce a publishable study report.

### Non-dissemination of individual findings of qualitative studies

About a third (35.6% of 810) of respondents reported that at least one of their qualitative reports did not include all of the important individual findings. About 4.3% of all respondents reported that more than half of their reports lacked an individual finding that they considered important (see Table 5). Researchers’ estimates of the proportion of non-dissemination of important individual findings by other researchers were clearly higher than their estimates for non-dissemination of their own individual findings. The majority (69.5% of 843 respondents) estimated that other researchers had at least one study report where important individual findings were missing.

**Table 5:** Comparison of proportions of researchers’ non-dissemination of important findings and their estimates of proportions of non-dissemination of important findings of other researchers.

Proportion of studies	Proportion of researchers who did NOT include all important findings (by categories in column 1) % (n) (N = 810)	Proportion of researchers that estimated that study reports by other researchers did NOT include all important findings (by categories in column 1) % (n) (N = 843)
0%	64.4 (522)	3.3 (28)
1–20%	25.7 (208)	23.1 (195)
21–40%	5.6 (45)	21.5 (181)
41–60%	2.1 (17)	14.4 (121)
61–80%	1.5 (12)	8.1 (68)
81–100%	0.7 (6)	2.4 (20)
Not sure	Not applicable	27.3 (230)

### Reasons for non-dissemination of important individual findings as reported by researchers

Around a third of 279 respondents reported that comments from a peer reviewer or editor (31.9%), resource constraints (28.7%), and limited data quality (26.9%) were reasons for their own non-reporting of selected important individual findings. In their free text responses, many researchers attributed selective dissemination of individual findings to the word limits of journals. However, researchers also noted that they often consciously reported only selected individual findings that suited the scope of the article and dropped those that did not fit their article. Some researchers said that their selection was motivated by what they saw as likely to be publishable. Many researchers also reported that they had many findings from one study and consequently some of them planned to publish more than one paper from a single study.

### The viewpoint of editors and peer reviewers

We asked editors to rate how strongly individual reasons affected their decision to reject manuscripts of qualitative studies. Reasons that the vast majority of the 133 participating editors “(strongly) agreed” with were: insufficient study quality (93.1%), insufficient reporting quality (90.9%), and a mismatch between the topic or findings of the study and the prioritized content by the journal or the general scope of the journal (90.9%). Furthermore, most editors “(strongly) agreed” that peer reviewers’ recommendation to reject a manuscript (78.3%), a lack of consideration of the journal’s instructions for authors (62.3%), and data that were too old (49.6%) affected their decision to reject a manuscript. Fewer said that they “(strongly) agreed” that the geographic focus of the manuscript (27.7%) or difficulties in identifying appropriate peer reviewers (20.0%), were reasons for rejecting a manuscript of a qualitative study. Lastly, 16.2% reported that they “(strongly) agreed” that a manuscript of a qualitative study would be rejected because it was seen as likely to generate few citations. In their free-text responses to this question, editors also stressed that poor reporting quality affected their decision to reject a manuscript of a qualitative study, but that findings that were not novel or did not add value to science were more important reasons.

The majority of the 682 participating peer reviewers said that they “(strongly) agreed” that insufficient study quality (95.0%) and reporting quality (95.0%) affected their recommendation to reject a manuscript of a qualitative study. Most peer reviewers reported that they “strongly agreed/agreed” that a mismatch between the manuscript’s topic and the journal’s prioritized scope (71.6%), as well as suspected incomplete reporting of findings (63.9%), data that were too old (44.3%) and a lack of consideration of the journal’s instructions for authors (37.1%), affected their recommendation to reject a manuscript. Only 18.2% of the peer reviewers “(strongly) agreed” that the geographical area that the manuscript reported on was a reason for rejection. Table 6 lists aspects of current journal publication policies and processes that editors and peer reviewers thought might impact on dissemination bias in qualitative studies. A rather similar proportion of editors (58.7%) and peer reviewers (75.6%) reported to view journal recommendations on manuscript length to impact on the fullness of reporting of findings from qualitative studies. On the other hand, the familiarity of peer reviewers with certain types of research was reported by considerably fewer editors (28.9%) than peer reviewers (79.4%) to impact on the full publication of qualitative studies.

**Table 6:** Respondents’ opinions on aspects of current publication policies and process that contribute to non-dissemination of qualitative research studies and/or findings.

Opinion	Editors % (n) (N = 121)	Peer reviewers% (n) (N = 680)
Journal instructions to authors are seen to exclude some types of qualitative research	62.8 (76)	48.7 (331)
Journal recommendations on manuscript length impact on the fullness of reporting of findings from qualitative research	58.7 (71)	75.6 (514)
Editors disfavor certain types of research studies	51.2 (62)	54.3 (369)
Editors are less familiar with certain types of qualitative research	40.5 (49)	59.6 (405)
Editors are less interested in qualitative research on some topics	40.5 (49)	51.2 (348)
Editors are less interested in qualitative research from some geographic regions or areas	33.9 (41)	21.9 (149)
Peer reviewers disfavor certain types of research studies	30.6 (37)	46.3 (315)
Peer reviewers are less familiar with certain types of qualitative research	28.9 (35)	79.4 (540)
Peer reviewers are less interested in qualitative research on some topics	20.7 (25)	56.3 (383)
Peer reviewers are less interested in qualitative research from some geographic regions or areas	17.4 (21)	22.4 (152)
Other reasons (free text responses)	17.4 (21)	10.3 (70)

### Authors of QES

We were also interested in the experiences and views of authors of QES. Of 840 respondents, 429 had conducted at least one QES, either as the lead author or as co-author (51.1%). While working on their QES, around half of 423 respondents said that they “always” or “often” considered dissemination bias throughout the whole process (48.1%), or when searching for literature (52.2%), when synthesizing and interpreting the findings (50.6%), and when considering the limitations of the synthesis (57.3%). The proportion of respondents who “rarely” or “never” considered dissemination bias in these stages ranged from 17.7% (when considering the limitations of the findings) to 23.5% (throughout the process).

### Attitudes towards consequences of dissemination bias in qualitative research

We asked all participants, regardless of their roles (researcher, author of QES, peer reviewer, editor), for their opinion about the potential consequences of dissemination bias. Of 800 respondents, 83.1% “(strongly) agreed” that non-dissemination and possible resulting dissemination bias might undermine the willingness of funders to support qualitative research in the future. 76.6% “(strongly) agreed” that the failure to disseminate qualitative research would undermine the willingness of researchers to conduct qualitative research, and that non-dissemination might provoke mistrust in qualitative research among health care professionals (77.5%), policy makers (76.8%) and the general public (48.8%). In addition, 72.6% and 71.2% respectively “(strongly) agreed” that non-dissemination might lead to inappropriate health policy and inappropriate health care.

### Attitudes on measures to decrease dissemination bias in qualitative research

Of 803 respondents, 33.4% agreed that *all* individual findings from qualitative research should be published, whereas 44.6% did not agree to that, and 22.0% said they were unsure. The majority of 792 respondents stated that authors should have the main responsibility for deciding whether they want to publish their findings (56.8%), which of their findings to publish (50.8%), and the most appropriate publication format (69.6%). In contrast, very few respondents said that funders should have the main responsibility for deciding whether findings are published (7.2%), which findings to publish (3.9%), and the most appropriate publication format for findings (7.6%). A joint decision about these issues between authors and funders was supported by 29.2% of the respondents, and 21.8% mentioned that other stakeholders (e.g., research participants) should have an important influence on decisions regarding the publication of qualitative research (multiple answers possible).

Of 794 respondents, 40% or more reported that they were unsure or had no opinion concerning measures to decrease dissemination bias. A large proportion, however, mentioned that the registration of qualitative studies in a publicly accessible registry (39.3%) and/or the publication of findings in a publicly accessible study registry (43.5%) might decrease the risk of dissemination bias. 17.8% and 16.5%, respectively, thought neither registration of a study nor a public repository for results would decrease the risk of dissemination bias.

In free text responses, respondents reported that they were concerned about the methodological quality of qualitative studies. Some responses indicated that registries might be useful in improving the quality of qualitative research conducted: “It would also discourage bad quality qualitative research, as people would have to think through at least some basics before they embark on a project.” Others mentioned that registries were not well suited to enhancing study quality and reducing dissemination bias. Other factors were identified as having a greater influence on dissemination bias: “It is time that is the major factor in writing up study findings and a register would not address this.” Respondents said that they liked the idea of a registry because it could give an overview of what research was being conducted and could give access to findings from studies that remained unpublished.

## Discussion

In our survey, over half of researchers reported that one or more of their qualitative studies had not been published in a peer-reviewed journal (≥1 study not published: 68.1%) or another publicly accessible format (≥1 study not published: 51.7%). One third reported that important individual findings were missing in one or more of their published reports (35.6% n = 288). Researchers reported that the most important reasons for non-dissemination were that publication was still planned, (35.7% n = 147), resource constraints, (35.4% n = 146), and rejection from journals (32.5% n = 134). Editors and peer reviewers reported that the methodological quality and reporting quality of qualitative studies were the main reasons for rejecting manuscripts. Most respondents reported that they agreed that non-dissemination of qualitative research has negative impacts on health research (76.6% n = 614), health policy (72.6% n = 581) and health care (71.2% n = 572). Researchers reported to have mixed views about whether publicly accessible repositories for registration of qualitative studies and dissemination of qualitative study findings might decrease dissemination bias.

When compared to their own reported publication practices, respondents estimated a higher proportion of not published/disseminated qualitative research among other researchers. While survey respondents often overestimate the extent to which they act in a socially desirable way, they may give more realistic estimates about other people’s behavior [21]. While both estimates might be biased towards over- or underestimation, our findings may indicate that the actual proportion of qualitative research that is not disseminated in any publicly accessible format is probably higher than the self-reported estimates of the survey respondents. As is the case for quantitative, clinical research, where about half of studies are not published in a scientific journal [22–24], the proportion of qualitative studies not published in peer-reviewed journals seems to be substantial. It is possible that non-dissemination in qualitative research is a larger problem than in quantitative, clinical research.

A sizeable proportion of researchers associated the non-dissemination of their qualitative studies with reasons also commonly reported by researchers in quantitative research and clinical trials [25]. In both environments, a lack of resources and especially a lack of time, and the fact that the publication of the study was not intended, played major roles. Researchers in quantitative biomedical research also mentioned journal editors, peer reviewers and the publication process as barriers to study publication [17]. In our study, the proportion of unpublished qualitative and quantitative studies, respectively, and their authors’ reasons for non-publication, are the main aspects that can be compared between these two types of research. Other features, including for example the description of the direction of study findings, differ substantially between qualitative and quantitative research and evidence on these aspects is lacking.

The responses demonstrate that editors’ and peer reviewers’ opinions seem to differ regarding the different aspects that impact on the full reporting of qualitative studies and their individual findings. Our survey could not determine to which degree the non-dissemination is influenced by each group of stakeholders. However, the responsibility of ensuring that all research is properly disseminated needs to be recognized and embraced jointly by researchers, editors and peer reviewers, and other stakeholder groups such as funders, as has been suggested for clinical trials [26], to improve unfavorable publication practices in science.

In the context of QES, systematic searches for published evidence are typically conducted in electronic journal databases [8]. Qualitative studies published or presented in books, reports, at conferences or in social media might in fact be publicly accessible, but likely missed in a standard systematic literature search. Consequently, in the context of QES, non-dissemination of qualitative studies might be illustrated more realistically by the proportions not published in peer-reviewed journals, because peer-reviewed journal publications seem to be accessible more easily than grey literature. While 80% of the responding authors of QES reported that they often or always considered dissemination bias during the research process—and we noted some examples of QES in which various forms of dissemination bias were addressed [27–29]—many QES that we are aware of rarely document or discuss dissemination bias. The causes of this discrepancy between the responses received in our survey and actual scientific conduct is not clear.

### Strengths and Limitations

Through our survey, we were able to reach an international audience of researchers that disseminate their findings in more than 30 different languages, acting as researchers, journal editors and peer reviewers as well as authors of QES. Therefore, a considerable strength of this study is its large sample size and broad variety of respondents in terms of current country of work, main publication language and age. Another strength is the fact that we supplemented closed questions with free text responses.

We applied different strategies to limit bias in our study. We facilitated honest responses by not collecting personal identifiers in connection to the survey responses and stating clearly that no individual taking the survey could be identified. Furthermore, we provided neutral information about the aim of our study and avoided communicating hypotheses that might have influenced the participants’ answers. We view the range of responses that we received as an indicator of the low likelihood of social desirability bias. Nonetheless, researchers reported that they had published rather high proportions of their studies and researchers with experience in conducting QES reported a high degree of attention towards dissemination bias within QES, despite the fact that the issue is rarely discussed in the QES literature. Both could indicate that participants might have tended towards socially desirable answers. Although this may be less dominant in surveys than in interviews [30], social desirability often entails over- or underreporting by participants. Additionally, survey responses might not accurately reflect the proportions of non-dissemination of studies and its reasons due to recall bias which is likely to have affected our findings as well.

We asked respondents to exclude research projects that they conducted/supervised in the context of scholarly degrees, such as masters’ or PhD theses, which might be a limitation of this study. Another potential problem is that respondents may have interpreted frequently used terms differently, and consequently answered the survey questions differently; we did not include precise definitions of the terms “study”, “finding”, “peer-reviewed journal”, “publicly accessible” etc.

Despite the large sample size and broad variability of respondents, the external validity of our results might be limited. One reason for this could be due to our non-probabilistic sampling methods and self-selection bias in who completed our survey. We identified survey participants through an electronic database (7.8%) and through our professional networks. The response count from these two sources shows that we recruited a greater number of people through our professional networks and snowball sampling (92.2% of respondents), which are not allocable to either of these sampling methods due to aspects within the design and functioning of the online survey. This may have increased the proportion of respondents who shared our interest in the issue at hand and who might have strong opinions—and thus could have influenced our findings. Moreover, there might have been considerable overlap of the people invited to our survey through the different mailing lists, because we were focusing on gathering information from people with experience in the field of qualitative research and chose the invitation channels accordingly. Furthermore, the availability of the survey online might have excluded people in settings where internet access is difficult, particularly for large volumes of data. Despite the high number of responses and a variety of themes in free text responses, we may also have inadvertently missed including researchers with different experiences and attitudes towards dissemination bias in qualitative research. While we were able to reach participants from 66 different countries, fewer than 10% of our respondents were based in South America, Africa or Asia, whereas almost half were from Europe, and one third based in North America. The views of stakeholders in regions such as South America and Africa are therefore likely to be underrepresented in our findings. Due to these potential biases we are reluctant about making strong inferences from our study sample to the general population of qualitative researchers, editors and peer reviewers, and advise a cautious interpretation of our findings. Our study does not support any strong recommendations or obvious solutions to decrease dissemination bias in qualitative research. Based on our findings and findings from studies on dissemination bias in quantitative research, preliminary suggestions to decrease the extent of non-publication of qualitative research are that funders could allocate resources specifically to the publication of a study or make a certain proportion of funding available only after the publication of the study. Editorial policies might account for differences between types of research to allow for appropriate reviews of studies. Researchers might, where appropriate, consider the quality of their methods and reporting in their studies. More detailed research on dissemination bias in qualitative research and possible approaches to reduce it is needed. Research on the nature and extent of dissemination bias in different contexts is another important topic for future studies.

Our survey explored the issue of non-dissemination and dissemination bias in qualitative research based on a large sample of researchers, editors and peer reviewers. Overall, we found that the proportion of non-dissemination in qualitative research is substantial, and that several stakeholder groups play an important role in this regard. Non-dissemination, and resulting dissemination bias, was not seen as merely a theoretical problem but was seen as having important impacts on health care research, practice and policy.

Our survey focused on the issue of non-dissemination of qualitative research and the general concept of dissemination bias in qualitative research. We did not examine—and are therefore unable to draw conclusions about—the *systematic* non-dissemination of qualitative evidence: ‘dissemination bias’. Future research should explore questions regarding dissemination bias in qualitative research due to the nature of findings, language of dissemination etc.

## Appendix

### Mailing lists that were utilized to distribute invitations to the survey

Authors, peer reviewers and editors of the Journal of Advanced Nursing: ca. 3000 recipients

Members of “JISCMAIL: Email discussion lists for the UK Education and Research communities” list ‘Evidence-based health’: ca. 1500 recipients

Members of “JISCMAIL: Email discussion lists for the UK Education and Research communities” list ‘Advice and Support in QUalitative evidence Synthesis (ASQUS)’: ca. 200 recipients

Health Information for All by 2015 (HIFA2015) discussion lists: ca. 8200 recipients

GRADE-CERQual mailing list: ca. 81 recipients

## Supporting Information

S1 File Supporting information file 1. (DOCX)

S2 File Supporting information file 2. (XLS)
