# Supplementary material for: Extent, Awareness and Perception of Dissemination Bias in Qualitative Research: An Explorative Survey
Source: PLoS One. 2016 Aug 3;11(8):e0159290. doi: 10.1371/journal.pone.0159290 (PMC4972302; doi:10.1371/journal.pone.0159290)
Supplement: S1 File — (DOCX) [file pone.0159290.s001.docx]

**Supporting information file 1: Questionnaire about non-dissemination of qualitative research**

1. Have you been research active in the past 5 years? (if no skip to question 10)
   - Yes
   - No

**Experience in conducting qualitative research and reporting the findings of qualitative research**

**In the following questions we would like to get to know more about your experience in conducting qualitative research**

1. How many primary qualitative research projects or projects with a qualitative component (excluding systematic reviews, evidence syntheses and meta-analyses) have you ever completed either independently or as part of a team? [Please give your best estimate]
   - 0 (if 0 skip to Q10)
   - 1-5
   - 6-10
   - 11-15
   - 16 or more
2. For the qualitative research projects you conducted, what proportion of has **not** resulted in a **peer-reviewed journal publication**? Please give your best estimate
   - 0%
   - 1-20%
   - 21-40%
   - 41-60%
   - 61-80%
   - 80-100%
3. For the qualitative research projects you conducted, what proportion of has **not** resulted in any write-up or report of findings that is publicly accessible? [Please give you best estimate]
   - 0% [if 0 skip to Q8]
   - 1-20%
   - 21-40%
   - 41-60%
   - 61-80%
   - 81-100%

**Dissemination practice in qualitative research**

**The next few questions ask about your practical experience in disseminating the reports or findings from qualitative research projects in which you were involved**

1. For the qualitative research project which did not result in any publicly accessible write up or report, which of the following best describes the reasons for non-dissemination? [If there is more than one project, think of the one you recall best. Tick all that apply]
   - Because the paper we wrote was rejected by one or more journals and we gave up
   - Because we never completed the study
   - Because we did not have enough resources for study publication (including time, money, staff etc.)
   - Because a publication was not intended
   - Because a publication is still intended
   - Because data were “thin” and themes were not saturated
   - Because we were not satisfied with the study quality
   - Because we thought findings were too controversial, in view of current knowledge in the field
   - Because the findings did not confirm our assumptions and concepts
   - Because language-issues made publication difficult
   - Because of disagreement among co-authors with regard to the publication
   - Not applicable

Other, internal reasons (please elaborate)

1. Were any external reasons relevant for non-dissemination of your qualitative research? [If there is more than one project, think of the one you recall best. Tick all that apply]
   - Because other researcher teams published papers with similar findings before we completed the study or wrote the paper
   - Because we thought journals were unlikely to accept the paper
   - Because the sponsors were not interested in publishing the findings
   - Because the sponsors prevented us from publishing the findings
   - Because the funder’s minimum reporting requirements were too high
   - Not applicable / There were no external reasons
   - Other external reasons (please elaborate)
2. For qualitative research projects in which you were involved, did any of the report(s) or paper(s) not include findings that you considered to be important? If yes, please give your best estimate of the proportion of these reports for which this is the case.
   - 0% (if 0 skip to Q9)
   - 1-20%
   - 21-40%
   - 41-60%
   - 61-80%
   - 81-100%
3. Which of the following best describes the reasons why specific important study findings were not included in reports or papers? [Tick all that apply]
   - Because of comments from a peer reviewer / journal editor
   - Because of comments from an investigator involved in the project
   - Because of disagreement among co-authors regarding the publication
   - Because we thought the data quality for the findings was insufficient
   - Because we thought the findings were too controversial, in view of current knowledge in the field
   - Because we thought the findings were of insufficient relevance
   - Because we did not have enough resources to write up the findings
   - Because we were unable to explain the finding(s) or did not consider the finding(s) to be clear
   - Other / not applicable (please specify) [free text]
4. Other than peer-reviewed journals, where else do you publish your qualitative research (e.g., social media, books, conference proceedings etc.)?
   - [free text]
5. In your view, what proportion of qualitative research projects conducted by other professional researchers, does not result in any write-up or report that is publicly accessible? Please give your best estimate
   - 0%
   - 1-20%
   - 21-40%
   - 41-60%
   - 61-80%
   - 81-100%
   - Not sure
6. In your view, what proportion of qualitative research reports by other researchers did not include all important study findings (e.g., only some findings or themes were published)?
   [Please estimate the proportion of study reports with incomplete representation of findings (%)]
   - 0%
   - 1-20%
   - 21-40%
   - 41-60%
   - 61-80%
   - 81-100%
   - Not sure
7. How many systematic reviews or evidence syntheses of qualitative studies have you conducted as a lead or co-author?
   - 0 (if 0 skip to question 14)
   - 1-5
   - 6-10
   - 11-15
   - 16 or more

**For authors of systematic reviews or evidence syntheses of qualitative studies**

1. How often did you consider dissemination bias while working on your systematic review(s) or evidence syntheses with qualitative research components?

| Review stage | Always | Often | Sometimes | Rarely | Never |
| --- | --- | --- | --- | --- | --- |
| Throughout the review process |  |  |  |  |  |
| When searching for literature |  |  |  |  |  |
| When synthesizing and interpreting findings |  |  |  |  |  |
| When considering the limitations of the review |  |  |  |  |  |

1. Are you an editor for a health- or social care related publication outlet (e.g., journal)? (if no, skip to question 17)
   - Yes
   - No

**Dissemination of qualitative research: the viewpoint of journal editors**

For editors

1. Please indicate the extent to which you agree that the following affect your decision **as** **an editor** to reject papers of qualitative research?

|  | Strongly agree | Agree | Undecided | Disagree | Strongly disagree |
| --- | --- | --- | --- | --- | --- |
| The topic / findings does not fit in the prioritized scope of the journal |  |  |  |  |  |
| The paper reports on a geographic area that is not a priority for the journal |  |  |  |  |  |
| The data were collected too long ago |  |  |  |  |  |
| The reporting quality of the paper is not sufficient |  |  |  |  |  |
| The study quality is not sufficient |  |  |  |  |  |
| The authors neglect the journal’s instructions for authors |  |  |  |  |  |
| It is difficult to identify appropriate peer reviewers |  |  |  |  |  |
| The peer reviewers recommend a rejection |  |  |  |  |  |
| The article is unlikely to generate many citations |  |  |  |  |  |
| Other, please elaborate |  |  |  |  |  |

1. In your experience as a journal editor, what aspects of your current journal publication policies and process might impact on dissemination bias in qualitative research? [Tick all that apply]
   - Journals instructions to authors are seen to exclude some types of qualitative research
   - Journal recommendations on manuscript length impact on the fullness of reporting of findings from qualitative research
   - Editors disfavor certain types of research studies
   - Editors are less familiar with certain types of qualitative research
   - Editors are less interested in qualitative research on some topics
   - Editors are less interested in qualitative research from some geographic regions or areas
   - Peer reviewers disfavor certain types of research studies
   - Peer reviewers are less familiar with certain types of qualitative research
   - Peer reviewers are less interested in qualitative research on some topics
   - Peer reviewers are less interested in qualitative research from some geographic regions or areas
   - Other [Open text question]
2. Have you ever acted as a peer reviewer for a scientific journal? (If no skip to Q 20)
   - Yes
   - No

For Peer Reviewers

Dissemination of qualitative research: the viewpoint of peer reviewers

1. Please indicate the extent to which you agree that the following affect your decision **as** **a peer reviewer** to recommend rejection manuscripts of qualitative research?

|  | Strongly agree | Agree | Undecided | Disagree | Strongly disagree |
| --- | --- | --- | --- | --- | --- |
| The topic / findings does not fit in the prioritized scope of the journal |  |  |  |  |  |
| The paper reports on a geographic area that is not a priority for the journal |  |  |  |  |  |
| The data were collected too long ago |  |  |  |  |  |
| The study quality is not sufficient |  |  |  |  |  |
| The reporting quality of the paper is not sufficient |  |  |  |  |  |
| Because of suspected incomplete reporting of relevant findings |  |  |  |  |  |
| The authors neglect the journal’s instructions for authors |  |  |  |  |  |

- - Other, please elaborate:

1. In your experience as a peer reviewer, what aspects of publication policies and process might impact on dissemination bias in qualitative research? [Tick all that apply]
   - Journal instructions to authors exclude some types of qualitative research
   - Journal recommendations on manuscript length impact on the fullness of reporting
   - Peer reviewers disfavour certain types of research studies
   - Peer reviewers are less familiar with certain types of qualitative research
   - Peer reviewers are less interested in qualitative research on some topics
   - Peer reviewers are less interested in qualitative research from some geographic regions or areas
   - Editors disfavour certain types of research studies
   - Editors are less familiar with certain types of qualitative research
   - Editors are less interested in qualitative research on some topics
   - Editors are less interested in qualitative research from some geographic regions or areas
   - Other [please specify]

**Attitudes on dissemination bias in qualitative research [for all respondents]**

**Here we would appreciate further details on your experience and attitudes regarding the dissemination of qualitative research**

1. In your opinion, to what extent do you agree or disagree with the following? The failure to disseminate qualitative research may:

|  | Strongly agree | Agree | Undecided | Disagree | Strongly disagree |
| --- | --- | --- | --- | --- | --- |
| Undermine the willingness of researchers to conduct qualitative research |  |  |  |  |  |
| Undermine health- or social care professionals’ trust in qualitative research |  |  |  |  |  |
| Undermine policymakers’ trust in qualitative research |  |  |  |  |  |
| Undermine the willingness of funders to support future qualitative research |  |  |  |  |  |
| Undermine the public’s trust in health research |  |  |  |  |  |
| Result in inappropriate health policy |  |  |  |  |  |
| Result in inappropriate health care |  |  |  |  |  |
| Other, please specify |  |  |  |  |  |

1. Do you agree that **all** findings from qualitative research projects should be published?
   - Yes
   - No
   - Unsure
2. In your opinion, who should mainly influence whether qualitative research projects should be published? (tick all that apply)
   - Authors should mainly influence whether they want to publish their findings
   - Authors should mainly influence which of their findings are published
   - Authors should mainly influence the most appropriate publication formats for their findings (journal, report, etc.)
   - Funders should mainly influence whether findings are published
   - Funders should mainly influence which of the findings are published
   - Funders should mainly influence the most appropriate publication formats for findings (journal, report, etc.)
   - Funders and authors should make all decisions together
   - Other stakeholders (e.g. research participants) should mainly influence decisions regarding the publication [please specify]
3. Please list the factors that, in your opinion, impact on whether qualitative research projects are published in a form that is publicly accessible (e.g. in a report or journal publication or on a website)?
   - Text box

**Measures to decrease risk of dissemination bias in qualitative research**

**Here we would like to gather your opinions on measures to improve the potential risk of dissemination bias in qualitative research.**

1. In your view, how would the **registration of qualitative research projects** in a public registry where the research aims and methods are publicly accessible impact on dissemination bias?
   - It would decrease the risk of dissemination bias
   - It would not decrease the risk of dissemination bias
   - I am not sure/no opinion
   - My comments on this issue: [text box]
2. In your view, how would the publication of **the findings** (or a summary of the findings) of qualitative research studies in a public study registry impact on dissemination bias?
   - It would decrease the risk of dissemination bias
   - It would not decrease the risk of dissemination bias
   - I am not sure/no opinion
   - My comments on this issue: [text box]
3. Would you like to leave any other thoughts or comments on the issue of dissemination bias in qualitative research?

**General information**

**Almost done! Please answer four additional questions.**

1. What is your age?
   - [text box]
2. What is your gender?
   - Male
   - Female
   - Other
3. What is your main country of work?
4. In which language(s) do you communicate findings from qualitative research? (Please list all that apply)

You have completed the survey! Thank you very much. Your participation will certainly help in answering our research aims.

If you are interested in receiving further information about our research, please subscribe to our mailing list. We will inform you about major progress in this study via e-mail. Please note, that your e-mail address will be stored separately from your survey responses so that they cannot be linked.

To subscribe to the mailing list, please copy and paste the following link to your browser:

<http://lists.cochrane.de/mailman/listinfo/disbiquare>
